# The Sufferings of Christ

**Published:** 1888-03-31

**Authors:** 


### Words of Consolation.

[Communications for this column should be addressed "Chaplain," care of the Editor of The Hospital, who will gratefully welcome any suggestions or inquiries from matrons, nurses, and the staff generally.]

### The Sufferings of Christ.—LXXI.

*For Reading to the Sick.*

When we compare our blessed Lord's passion and death so closely with the consequences due to sin, there is one idea we must carefully guard against—namely, that the Father in wrath visited the penalty for sin upon the Son : as if the Son were compelled to bear the curse, or otherwise under no condition should the door of Heaven be opened. No, God was not satisfied nor delighted with the terrible sufferings of His Son. Rather the satisfaction was found in the complete obedience under suffering ; obedience first of all through a life of temptation and trial, then under extreme mental and physical agony, even unto death. But why, it is sometimes asked, must there have been so much suffering if God did not demand it as a kind of condition for our redemption? The answer may perhaps be found more in the voluntary submission of the Son than in any supposed conditions dictated by the Father. It pleased our Lord to identify Himself so completely with our suffering humanity, to be so entirely one with us, sin only excepted, that to have suffered less than He did would have been to detract something from such a union. The Head of the race, the Captain of our salvation, must be able to say, not only to the whole human family redeemed, but to each member : "Lo! here in your own world, and in your very nature, I have borne your sin, and just what that sin can inflict upon you. You have seen what grievous injury sin can inflict upon an innocent person. You have seen how I accepted that injury — at what a cost; at what loss to myself. You see that through dying unto sin once I have pointed as well to sin's consequences hereafter. If after such a manifestation of love in suffering you can continue to be the friend of sin, what more can be done for you? Here you have the highest motive for dying unto sin. Kill it because it killed Me. Mortify it for My sake. Let the love of the Atonement become the spring of your life. Mortify it for your own sake, lest it eventually bring you to a worse death than even I endured." So much we venture by way of recapitulation; because we shall now proceed to inquire what security is to be found in Christ for continuing in the way pointed by the Cross. There freedom from guilt, however blessed the idea of complete forgiveness may sound to a once sin-burdened soul, is no liberty, unless we go on to destroy the principle of sin which is within us. You may be freed from guilt, but if the principle or root from which that guilt proceeded be allowed to remain in your being the guilt will assuredly set in again. We only say this in order that you may clearly distinguish between sin as a debt and sin as a disease. The death of Christ cancels the debt for all who are brought into covenant with Him certainly. But what does it do besides towards eradicating the disease? What does it do towards rooting out evil principle and supplying the inner man with the grace of a new and abundant life. We want to show you next that the essence from the Cross of Christ supplies not only a motive but a security for holiness. He died that we might be forgiven, but through His death and resurrection He has also given a pledge for a new life being bred and sustained within the souls of His elect. With this question is concerned one of the deepest mysteries of His dissolution. We use the word dissolution advisedly, because we are proceeding to discuss the question of life rather than the terrors of desertion and death,

